# Why the hypothesis of embryo selection in IVF/ICSI must finally be reconsidered

**DOI:** 10.1093/hropen/hoaf011

**Published:** 2025-03-20

**Authors:** Norbert Gleicher, Sonia Gayete-Lafuente, David H Barad, Pasquale Patrizio, David F Albertini

**Affiliations:** Center for Human Reproduction (CHR), New York, NY, USA; Foundation for Reproductive Medicine, New York, NY, USA; Stem Cell and Embryology Laboratory, Rockefeller University, New York, NY, USA; Medical University of Vienna, Vienna, Austria; Center for Human Reproduction (CHR), New York, NY, USA; Foundation for Reproductive Medicine, New York, NY, USA; Center for Human Reproduction (CHR), New York, NY, USA; Foundation for Reproductive Medicine, New York, NY, USA; Center for Human Reproduction (CHR), New York, NY, USA; Department of Obstetrics, Gynecology and Reproductive Sciences, University of Miami, Miller School of Medicine, Miami, FL, USA; Center for Human Reproduction (CHR), New York, NY, USA; Bedford Research Foundation, Bedford, MA, USA

**Keywords:** IVF, embryo selection (ES), ES methods, cumulative pregnancy rate, cumulative live birth rate, infertility, artificial intelligence (AI)

## Abstract

Embryo selection (ES) during IVF is expected to select the ‘best’ embryo(s) from among a cycle’s embryo cohort and has been a core concept of IVF for over 40 years. However, among 36 492 articles on ES in a recent PubMed search, we were unable to locate even a single one questioning the concept that, beyond standard oocyte and embryo morphology, ES has remained an unproven hypothesis. In unselected patient populations, attempts at ES have universally, indeed, failed to improve cumulative pregnancy and live birth rates. The only benefit ES appears to offer is a marginal shortening in time to pregnancy, and even this benefit manifests only in best-prognosis patients with large oocyte and embryo numbers. Excluding *in vitro* maturation efforts, oocytes, once retrieved, and their resulting embryos have predetermined finite cumulative pregnancy and live birth chances that cannot be further improved. The hypothesis of ES has, however, remained a driving force for research and the introduction of a multitude of ‘add-ons’ to IVF. Enormous investments over decades in ES, therefore, should be better redirected from post- to pre-retrieval efforts.

## Introduction

Originally developed in natural cycles (Steptoe and Edwards, 1978), it quickly became apparent that IVF would advance from an experiment to a successful infertility treatment once combined with the use of gonadotropin stimulation (Jones *et al.*, 1982). IVF, furthermore, offered advantages over intrauterine inseminations by controlling the risks of multiple pregnancy (Gleicher *et al.*, 2000). Growing embryo yields from gonadotropin stimulations and lower embryo transfer numbers to prevent multiple births created a seemingly obvious incentive for embryo selection (ES), which over more than 40  years has consumed more research efforts and funding than any other IVF-related research. Yet to this day, it has failed to develop new cost-effective treatment options to improve IVF cycle outcomes.

**Figure 1.**
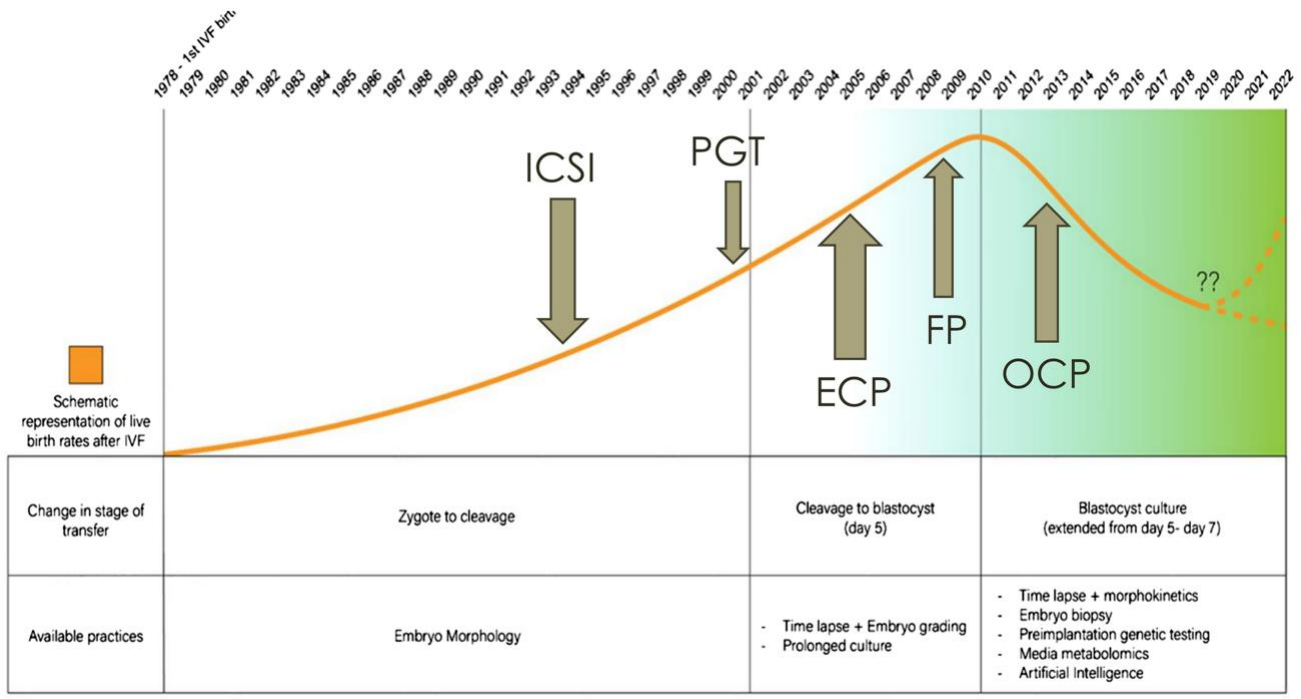
**Timeline of IVF live birth rates and key technological milestones (1978–2022).** Schematic illustrating the increase in IVF live birth rates per cycle between 1978 and 2022, highlighting key milestones that have changed the course of IVF practices as discussed in the text. Arrows demarcate the approximate years of the introduction of specific technologies that have impacted IVF practice patterns globally. These have included ICSI, preimplantation genetic testing (PGT), embryo cryopreservation (ECP), fertility preservation (FP), and oocyte cryopreservation (OCP). The lower half of the figure highlights the transition in the stage of embryo development at transfer and the shift in embryo selection strategies from 2000 to 2022. *Important note*: The construct of this figure is symbolic and does not reflect real-life numbers. The figure also does not reflect age changes in the patient population (though the median age of women starting cycles in the USA has been stable at around 36 years) and changes in the number of embryos transferred, which, due to increased utilization of elective single embryo transfer (eSET), has declined and clearly has contributed to the lower live birth rates but also lower rates of multiple pregnancies.

Using a term introduced around 2017 to describe new additions to routine IVF treatment cycles (Fig. 1) (Harper *et al.*, 2017), many so-called add-ons to IVF have indeed proposed to enhance ES. However, in most instances, they have failed to improve IVF outcomes (ESHRE Add-ons Working Group *et al.*, 2023) and, overall, have likely contributed to the worldwide declines over the last 20 years in live birth rates in association with IVF/ICSI (Gleicher *et al.*, 2015). Many such ES procedures, including routine blastocyst culture (BSC) and preimplantation genetic testing for aneuploidy (PGT-A), have become mainstays of IVF/ICSI practice (Gleicher *et al.*, 2021, 2022a,b; Glujovsky *et al.*, 2022). A recently published multicenter randomized controlled trial of time-lapse imaging (which has been introduced at extraordinary costs to many IVF laboratories around the world) has only strengthened this argument further (Bhide *et al.*, 2024).

As this Opinion explains in detail, ES to this day has remained an unvalidated hypothesis. Based on biological considerations alone, it should have been recognized much earlier as a failed hypothesis. That this did not happen was at least partially the consequence of too much financing made available by industry and equity investors in pursuing ES, with the latest wave represented by the innumerous artificial intelligence (AI) products currently under investigation and/or already commercially claiming to have mastered ES, even though a very recent clinical trial was unable to demonstrate outcome differences between IVF cycles with embryos being judged by either manual morphology or deep learning (Illingworth *et al.*, 2024).

The belief in ES has been an essential component in capital markets viewing infertility treatments as a potentially lucrative growth industry and, therefore, as a continuous investment target which, especially over the last decade, has led to radical worldwide changes in the commercial fabric of the IVF field (Stein and Harper, 2021; Patrizio et al., 2022). Unsurprisingly, the utilization of revenue-generating new add-ons to IVF, consequently, has increased (Gleicher *et al.*, 2019; Glatthorn and Decherney, 2022).

This Opinion argues that, apart from standard morphological assessments of oocytes and embryos in the embryology laboratory during IVF cycles, the concentration of the IVF field on improving IVF cycle outcomes through ES has failed. Consequently, research efforts should be redirected.

## The principle of selection

Even in fertile couples, most reproductive failures occur early and in association with genetic instability of embryos (Jarvis, 2016) and, in patients with infertility, the physiological reproductive processes must be less efficient. Therefore, forces that shape fecundity in healthy, reproductively competent individuals, must be distinguished from what infertile populations face when they are subjected to fertility treatments, including IVF (Lubinsky, 2018). Effort to improve reproductive outcomes for infertile couples, consequently, must be differentiated from factors that contribute to natural conceptions.

Follicles and oocytes employ natural selection strategies. In natural cycles, in most cases, only one oocyte is ovulated and then fertilized; this means that more are recruited than, ultimately, are offered a chance of pregnancy. Nature’s selection strategies, therefore, have inspired IVF practice to obtain multiple oocytes. But the embarrassment of riches in achieved oocyte and embryo numbers has necessitated a selection process to identify the ‘best’ oocyte(s) and embryo(s).

Once it became apparent that basic embryo morphology was able to differentiate between embryos with poorer and better pregnancy and live birth chances, the hypothesis of ES won wide acceptance. A principal problem, however, arose with the presumption that ES could be further improved beyond that achieved with basic embryo morphology. The assumption was that ES would, like morphology, not only allow for the differentiation between ‘poorer’ and ‘better’ embryos but also would become numerically predictable of IVF cycle outcomes. As an additional side-benefit, ES then would also permit accurate deselection of ‘abnormal’ embryos from future use.

Though just an unvalidated hypothesis, ES was fully supported by leading professional societies, and, nevertheless, led to the worldwide routine utilization of several add-ons to IVF, with the two most consequential likely being routine blastocyst stage culture (Practice Committees of the ASRM and SART, 2018a) and PGT-A (Practice Committees of the ASRM and SART, 2018b). The most obvious add-on to demonstrate that ES cannot reliably distinguish between transferrable and non-transferrable embryos was probably PGT-A, which has now been demonstrated as failing to achieve this aim (Barad *et al.*, 2022a,b; Viotti *et al.*, 2023) and was recently formally recognized as such by the Practice Committees of the American Society for Reproductive Medicine (ASRM) and its daughter society, the Society for Assisted Reproductive Technology (SART) (Practice Committees of the ASRM and SART, 2024). Consequently, likely hundreds of thousands of embryos with decent pregnancy chances have been discarded over the last two decades and often are still being discarded.

Although the natural cumulative pregnancy chances obviously increase over time, it still took a recent study to re-emphasize that the number of retrieved oocytes and embryos produced in IVF cycles actually matters (Fanton *et al.*, 2023). It is, indeed, this basic observation that allows for the definition of ‘infertility’ as the failure to conceive within a statistically expected, age-specific time period. This observation of natural fecundity, of course, also informs fertility treatment-dependent pregnancy and live birth chances, reemphasizing the importance of oocyte and embryo numbers in assessing a patient’s cumulative pregnancy and live birth chances from single-cycle oocyte and embryo cohorts. It has been known for decades that live birth rates from individual oocytes in humans are very low, and between 2014 and 2020, the rate was not boosted despite steadily increasing utilization of ES-driven add-ons, including routine blastocyst-stage culture and PGT-A (Sabbagh *et al.*, 2023).

Simple math, therefore, suggests that every oocyte cohort and, therefore, every embryo cohort in an IVF cycle, represents a predetermined cumulative pregnancy and live birth chance for that cohort. Once the oocytes are retrieved, these cumulative outcome chances can only marginally vary, mostly depending on the quality of the embryology laboratory. Whichever embryo(s) is/are chosen first for transfer, therefore, does not affect the cumulative pregnancy and live birth chances at all. After basic embryo morphology, the only potential contribution of ES is a possible shortening of time to pregnancy—at most by a few months, and only in good-prognosis patients.

Here is how all of this relates to current IVF practice. As noted, ES started with assessments of cleavage stage morphology (Cummins *et al.*, 1986) and, through extended embryo culture to the blastocyst stage, was first alleged to improve IVF cycle outcomes in 1998 (Gardner *et al.*, 1998)—a fact that has ever since been widely overlooked is that the study participants had been highly selected as having a good prognosis. In addition, they then underwent yet another selection step through extended embryo culture to blastocyst stage, as poor-prognosis patients often no longer produce embryos that survive extended culture (Schoolcraft and Gardner, 2000).

For several reasons the study’s conclusions, therefore, were misleading for the following reasons: (i) the study subjects were highly selected good-prognosis patients, while the authors incorrectly concluded that the reported outcomes should apply to all IVF patients; (ii) the authors failed to note that blastocyst-stage culture did not increase cumulative live birth rates; (iii) poor-prognosis patients only rarely produce embryos that reach the blastocyst stage; and (iv) the reporting IVF cycle outcomes with reference embryo transfer, implicitly actually selected against data from the poorer-prognosis patients who produced few or no blastocysts. As a result, the pregnancy and live birth outcomes were considerably inflated in the paper by Gardner *et al.* Recently, the last point was especially highlighted as a frequent statistical error made in reporting treatment outcomes for infertility treatments (Wilkinson, 2023), suggesting that consumers of our medical literature must be more critical.

Subsequent studies in unselected general populations uniformly were unable to demonstrate clinically relevant outcome advantages for extended embryo culture (Glujovsky *et al.*, 2022). Extended embryo culture, indeed, can only negatively influence the cumulative pregnancy chance of an IVF cycle’s embryo cohort. This will be the case when an embryo arrests in extended culture between the cleavage and blastocyst stages. However, if it was transferred at the cleavage stage, it could still result in a normal pregnancy/live birth. A negative effect on the chances of cumulative pregnancy or live birth can, of course, also be the result of poor embryology and/or other clinical practice insufficiencies.

That at least some embryos which fail to reach the blastocyst stage in even good embryology laboratories can produce normal pregnancies/deliveries if transferred at the cleavage stage has been demonstrated by publications reporting similar cumulative pregnancy and live birth rates for oocyte/embryo cohorts in single IVF/ICSI cycles or even demonstrating small outcome advantages for all-cleavage-stage over all-blastocyst-stage embryo transfers (Cameron *et al.*, 2020; De Croo *et al.*, 2022; Mengels *et al.*, 2024); however, this is disputed without evidence by many colleagues. Considering the obvious patient selection biases favoring poorer-prognosis patient undergoing cleavage-stage transfers and better-prognosis patients having blastocyst-stage transfers, overall, these outcomes must be viewed as mildly supportive of universal cleavage-stage transfer (De Vos *et al.*, 2016; Martins *et al.*, 2017). Even the first transfers of blastocyst-stage embryos in unselected general infertility populations were unable to demonstrate outcome benefits over cleavage-stage transfers (reviewed by Gleicher *et al.*, 2015).

Endorsed as such by ASRM (Practice Committees of the ASRM and SART, 2018a), ES through embryo culture to blastocyst stage is, nevertheless, now the routine embryo culture method in almost all IVF clinics in the USA, for all patients of all ages. Considering that good prognosis patients, representing on average at most 15–20% of patients in IVF clinics, already have the best pregnancy chances in IVF practice (except for those undergoing donor-recipient cycles), whether routine utilization of BSC makes either clinical or economic sense is, therefore, questionable.

Probably the second most popular ES technique is PGT-A, which is of course automatically linked to routine blastocyst-stage culture. This procedure, for several reasons, significantly reduces cumulative pregnancy and live birth chances (Kucherov *et al.*, 2023). False-positive PGT-A results, which to this day often prevent good embryos from being transferred, are currently still, likely, the most frequent reason. Moreover, since current PGT-A requires extended embryo culture to the blastocyst-stage to perform a trophectoderm biopsy and requires embryo cryopreservation (CP), PGT-A is associated with three independent causes for poorer pregnancy/live birth outcomes (PGT-A, BSC, and CP) which, of course, act additively.

That ES beyond basic embryology is unlikely to succeed, is also suggested by the field’s experience with closed incubation systems and time-lapse imaging. Like other add-ons touted to improve IVF cycle outcomes, these have so far failed to do so and are unable to outperform standard embryology (Kieslinger *et al.*, 2023). Whether added deep-learning-based AI (Kromp *et al.*, 2023) will add prognostic value to ES, also appears questionable because even AI requires a discernable significant and clinically relevant difference between the embryos, if such a difference is to be detected. Whether enough difference exists to warrant the effort is questionable (Harper *et al.*, 2017; Stein and Harper, 2021; Glatthorn and Decherney, 2022) and, indeed, to date, deep learning, compared to manual morphology-based ES in IVF, has failed to demonstrate non-inferiority in clinical pregnancy outcomes (Illingworth *et al.*, 2024) despite presenting a number of real-world inconveniences that warrant further evaluation (Kieslinger *et al.*, 2024).

## Clinical IVF practice

Figure 1 graphically demonstrates the approximate timelines for the two most important trends in IVF practice: the addition of add-ons and the length of time embryos are cultured *in vitro* before transfers. Over the history of IVF, the time in culture was steadily extended from the initial zygote-stage transfers and, selectively, early cleavage stage transfers to routine Day-3 cleavage stage transfer, then to cleavage- and blastocyst-stage transfers and, finally, to routine blastocyst-stage transfer. In parallel, the number of new tests and add-ons to IVF, including CP, has increased, with the costs, of course, passed on to patients through additional charges (Dolinko *et al.*, 2017). Yet, the worldwide live birth rates since 2010 have mostly declined (Gleicher *et al.*, 2019, 2022b; Glatthorn and Decherney, 2022; Maheshwari *et al.*, 2022; Richter and Richter, 2023).

The utilization of multiple add-ons to IVF will compound the adverse effects and also attract additional concerns. Extended embryo culture was, for example, also described to impose epigenetic stress, impacting the short- and long-term health of human offspring (Wilson and Wallingford, 2021; Wang *et al.*, 2022). The effects on epigenetic reprogramming of embryos were first described in animal models (Calle *et al.*, 2012) but have since also been reported in humans (Barberet *et al.*, 2021; Menezo *et al.*, 2022).

Under- and over-expression of DNA repair genes and cell cycle checkpoint control genes (Zheng *et al.*, 2005) are other concerns expressed, as are potential adverse influences from *ex vivo* oxidative stress and the need for maximal oxidative stress management originating from the culture environment and from the heightened metabolism exhibited as embryos approach the blastocyst stage. This likely involves changes in mitochondrial metabolism, the imposition and repair of double-strand DNA damage. Additionally, there are mechanisms at the 1–2 cell stage of development that are linked to embryo quality but may not deter development to the blastocyst stage (Liang *et al.*, 2023). Finally, extended embryo culture has also been associated with increased risks of preterm births and placenta previa (Alviggi *et al.*, 2018; Spangmose *et al.*, 2020).

ES via PGT-A is a binary decision for each embryo, declaring it either fit or unfit for embryo transfer (Gleicher *et al.*, 2021, 2022a,b). Its impact is, moreover, compounded by the need for blastocyst-stage culture and embryo CP, and the fact that blastocyst-stage culture is usually almost always linked with elective single embryo transfer, with almost all these additional add-ons independently associated with reduced pregnancy and live birth rates. In particular, PGT-A likely has the largest cumulative adverse outcome among all of the add-ons. If used at all then, ES would have to consider more multiplex-based platforms to assess embryos (Shah *et al.*, 2022).

The IVF field also only recently started to contextualize the importance of self-correction of aneuploidy in human embryos. Elimination of aneuploid cells occurs downstream from blastocyst-stage primarily only in the embryonic cell lineage (which produces the fetus). The extraembryonic cell lineage (forming the trophectoderm and, later, the placenta) demonstrates this ability only to a much lesser degree, as first demonstrated in the mouse (Bolton *et al.*, 2016) and more recently in human embryos (Yang *et al.*, 2021). The human placenta, therefore, even with fully euploid fetuses maintains aneuploidy until delivery (Coorens *et al.*, 2021). By taking embryo biopsies during PGT-A from the placental pre-cursors, the trophectoderm, and thus from the extra-embryonic cell lineage, the obtained ploidy results may not be reflective of the fetus. Moreover, even if they are labeled as aneuploid, the fetus may still self-correct downstream from blastocyst stage, when the biopsy was taken, resulting in a false-positive PGT-A diagnosis and, therefore, often non-use or disposal of embryos with a normal potential for pregnancy and live birth (Gleicher *et al.*, 2021, 2022a; Maheshwari *et al.*, 2022).

## Good eggs make good embryos

Add-ons are of special concern in poor-prognosis patients. This is well demonstrated by the surprisingly good live birth rates after transfers of ‘non-transferrable’ embryos, defined as ‘mosaic’ and even ‘aneuploid’ embryos, from PGT-A cycles (Barad *et al.*, 2022a,b; Viotti *et al.*, 2023). Most IVF clinics currently, however, still do not transfer and often still dispose of such embryos, thereby indisputably reducing a patient’s cumulative pregnancy potential. Cleavage-stage instead of blastocyst-stage embryo transfers in such patients would, overall, appear to offer better pregnancy and live birth chances in a precision medicine-based individualized medical imperative (Maheshwari *et al.*, 2016; Xiao *et al.*, 2019).

Growing follicles mature in waves (Kirillova *et al.*, 2021); yet in natural cycles only a single dominant follicle reaches ovulation, and it is widely assumed to yield the ‘best’ oocyte and generating the ‘optimal’ embryo. The corollary to this assumption is that ovarian stimulation produces ‘rescued’ and therefore obviously ‘inferior’ follicles and oocytes (Macklon and Fauser, 2000). The overall success of IVF, however, strongly suggests that this level of inferiority cannot be very substantial. IVF experience furthermore suggests that among those inferior oocytes, beyond standard morphology and at the same maturity grades, the heterogeneity may be only relatively minor.

Sibling embryos in a cohort can be expected to be more like each other than non-related embryos. This has been confirmed in carefully selected young oocyte donors who vary in their respective oocyte-to-live birth ratios between ‘better’ and ‘poorer’ oocyte donors (Martin *et al.*, 2010). Individuals, therefore, even at young ages, can have ‘better’ or ‘poorer’ oocytes. Classifications of embryos according to their ability to lead to pregnancy, therefore, are less dependent on the oocytes than on the patients themselves. In other words, embryo cohorts for a particular patient are more similar than embryo cohorts between patients. This represents yet another reason why ES in IVF cycles must be rather inefficient.

## How we got here and what to do about it?

Medicine’s first ethical mandate is to cause no harm; its second most important mandate is to act in the best interest of patients in assuring favorable risk–benefit ratios and/or cost–benefit ratios for all interventions: a point recently re-emphasized in association with ES through PGT-A (Mastenbroek *et al.*, 2021). Four questions surrounding the enigma of ES have dominated IVF practice to this day. (i) What embryonic stage after fertilization would most likely establish pregnancy? (ii) What morphological, biochemical, or genetic determinants would best identify embryos with the greatest chances of establishing pregnancy? (iii) How would success in ES strategies be evaluated? (iv) Is multiple or elective single embryo transfers the safest and most productive means of producing healthy offspring?

From what we have here presented, one can conclude that all four of these questions actually have the same answer. Only individualization of care, not based on findings in individual oocytes and/or embryos as ES proposes, but based on the overall characteristics of individual patients, will lead IVF practice toward ‘precision medicine’ and, through this step, toward the maximizing IVF cycle outcomes.

Some examples for such an approach have already been reported. Because the metabolism in follicles speeds up with female age (and in younger women with premature ovarian aging), premature luteinization of follicles occurs progressively earlier with advancing age (Wu *et al.*, 2015). Triggering ovulation independent of patient age (and functional ovarian reserve) approximately at a lead-follicle size of 18–22 mm (which has been a standard practice almost since inception of IVF) is therefore no longer sustainable (Wu *et al.*, 2018). In fact, advancing triggers to lead-follicle sizes as small as 10–12 mm has allowed women of advanced maternal age—of up to 48  years—to conceive in IVF cycles with autologous oocytes (Gleicher *et al.*, 2018). Moreover, advancing age was recently also demonstrated to affect the ability of oocytes at different ages to produce good quality embryos, with metaphase II oocytes declining and germinal vessel oocytes greatly improving in ability to produce good quality embryos (Nicholas *et al.*, 2023), thereby further confirming the need for strict individualization of when to trigger ovulation in IVF cycles based on individual patient characteristics. However, AI platforms attempting to optimize ovulation trigger decisions have so far not been very successful (Canon *et al.*, 2024; Pérez-Padilla *et al.*, 2024).

Recently, for the first time, Italian colleagues have highlighted the potential emotional consequences of ES. They have also raised psychological concerns by, for example, astutely noting the disappointment a patient might feel after failing to conceive with what was considered the ‘best’ embryo, leaving them with only ‘second best’ options (Spinelli *et al.*, 2024). It is time to stop searching for and referring to ‘best’ embryos.

## Authors’ roles

N.G. and D.F.A. developed the concept for this manuscript and wrote the initial draft. Both authors contributed equally to all aspects of this paper. D.H.B. and P.P. entered the discussion at a later point and contributed to subsequent versions of the paper. S.G.-L. addressed two of the revisions required by *Human Reproduction Open* reviewers and editors to generate the final version of the manuscript. All the authors approved of the final version of the paper.
